# Supplementary material for: Contamination detection in genomic data: more is not enough
Source: Genome Biol. 2022 Feb 21;23:60. doi: 10.1186/s13059-022-02619-9 (PMC8862208; doi:10.1186/s13059-022-02619-9)
Supplement: Supplementary file 1 — Additional file 1: Supplemental Notes. Comparison and benchmarking of six contamination detection tools. The note compares and benchmarks six of the 18 tools discussed in the article: CheckM, EukCC, Forty-Two, GUNC, Physeter and Kraken2. These programs are tested on artificial chimerical genomes containing both redundant and non-redundant as well as inter-domain contaminations. Typical command lines and output for each of these six tools are also provided. [file 13059_2022_2619_MOESM1_ESM.html]

# Cornet L. & Baurain D. (2022) Contamination detection in genomic data: more is not enough

# Supplemental Note 1

## 1. Singularity setup

This note benchmarks six different contamination detection programs on the same data. It covers the majority of the algorithms relying on a reference database. We further provide Singularity [63] definition files to help with the installation of these programs and ensure reproducibility of the reported results. These files are accessible through the Figshare repository https://doi.org/10.6084/m9.figshare.17705558.v1.

```
$ wget XXXX     # Python script for chimeric genome creation
$ wget https://figshare.com/ndownloader/files/32405687 -O Cornet-Baurain-contams.tgz
$ tar -xzf Cornet-Baurain-contams.tgz
Cornet-2022-GBIO-Figshare/
├── contams.def             # Singularity def file for programs part 1 (all programs except EukCC)
├── eukcc.def               # Singularity def file for programs part 2 (EukCC)
├── Chimeric-genomes.py     # Python script for chimeric genome creation
├── contam-labels.idl       # idl file use for Physeter and Forty-Two (from https://bitbucket.org/phylogeno/42-ribo-msas/)
├── life-tqmd-of73.dmnd     # DIAMOND blast database for Physeter (from https://doi.org/10.3389/fmicb.2021.755101)
├── life-tqmd-of73.gca      # List of GCA numbers for Physeter database (needed to enable the k-fold mode)
├── taxdump-20211206        # NCBI Taxonomy dump used across the study
└── README
```

Informations on how to use Singularity containers can be found here:

- https://sylabs.io/guides/3.0/user-guide/build\_a\_container.html
- https://sylabs.io/guides/3.0/user-guide/bind\_paths\_and\_mounts.html

We used Singularity v3.8.0 and containers were bound to working directories. To facilitate the reading of this note, Singularity-related commands were not transcribed; only commands driving the programs themselves are reported here.

## 2. Creation of chimeric genomes

Two chimeric genomes were created to systematically test genomic contamination detection programs, one including only bacterial sequences and one combining bacterial and eukaryotic sequences.

For the bacterial chimera, two uncontaminated bacterial genomes were selected as references and a chimeric genome mixing sequences from these two genomes was created using a custom script (see above). Both redundant and non-redundant contaminations were simulated, based on “exchanges” of core-gene coding sequences (CDS) after orthologous gene inference.

Briefly, `Prodigal` v2.6.3 (https://github.com/hyattpd/Prodigal) was used to predict proteins on the two uncontaminated genomes. `inst-abbr-ali.pl` v0.212670 (https://metacpan.org/dist/Bio-MUST-Core) was then used to format the names of the sequences. `OrthoFinder` v2.5.4 (https://github.com/davidemms/OrthoFinder) was used on these proteins files to infer orthologous groups. Groups with only one sequence from each genome (i.e., single-copy core genes) were randomly sampled either to replace (non-redundant contamination) or supplement (redundant contamination) the corresponding CDS from one genome (here named the *master genome*) with sequences from the second genome (here named the *sub-genome*).

### 2.1 Reference genome download

Two genomes were selected based on the results of our contamination analyses of NCBI RefSeq [45-46]. These genomes should have no contamination reported by CheckM [48] with a maximum of two contigs (e.g., one chromosome and one plasmid). Here, we used a Firmicutes (`GCF_000003645.1`) (https://www.ncbi.nlm.nih.gov/assembly/GCF\_000003645.1/) as the master genome and a (Gamma)Proteobacteria (`GCF_000007145.1`) (https://www.ncbi.nlm.nih.gov/assembly/GCF\_000007145.1/) as the sub-genome.

```
$ wget https://ftp.ncbi.nlm.nih.gov/genomes/all/GCF/000/003/645/GCF_000003645.1_ASM364v1/GCF_000003645.1_ASM364v1_genomic.fna.gz
$ wget https://ftp.ncbi.nlm.nih.gov/genomes/all/GCF/000/007/145/GCF_000007145.1_ASM714v1/GCF_000007145.1_ASM714v1_genomic.fna.gz
```

### 2.2 Protein prediction

`Prodigal` was used to predict the proteins, then definition lines were abbreviated with `inst-abbr-ali.pl`.

```
$ prodigal \
    -i GCF_000003645.1_ASM364v1_genomic.fna \
    -o GCF_000003645.1.out -a GCF_000003645.1.faa -d GCF_000003645.1.genes.fna
$ prodigal \
    -i GCF_000007145.1_ASM714v1_genomic.fna \
    -o GCF_000007145.1.out -a GCF_000007145.1.faa -d GCF_000007145.1.genes.fna
$ inst-abbr-ids.pl GCF_000003645.1.faa --id-regex=:DEF --id-prefix=GCF_000003645.1
$ inst-abbr-ids.pl GCF_000007145.1.faa --id-regex=:DEF --id-prefix=GCF_000007145.1
```

### 2.3 Orthologous group inference

`OrthoFinder` was used to infer the orthologous groups.

```
$ mkdir OF-indir
$ cp *.faa OF-indir/
$ orthofinder -t 10 -a 10 -f OF-indir/
$ mkdir OG
$ mv OF-indir/OrthoFinder/Results_*/Orthogroup_Sequences/*.fa OG/
```

### 2.4 Chimeric bacterial genome creation

We designed a Python script to automatically create the chimeric genomes: `Chimeric-genomes.py`. It takes as input the protein files for the master and sub-genomes, then replacement and addition of genes in the master genome are carried out based on single-copy core genes. Redundant and non-redundant contamination levels were both set at 5% of the number of proteins. These percentages correspond to the threshold values proposed in [64].

```
$ ./Chimeric-genomes.py GCF_000003645.1.faa GCF_000007145.1.faa --replacement=5 --redundancy=5
Master genome: GCF_000003645.1 contains 5382 genes.
Sub genome: GCF_000007145.1 contains 4292 genes.
269 master genes replaced by genes from sub genome: GCF_000007145.1
269 master genes duplicated by genes from sub genome: GCF_000007145.1
Master genome represents 88.1064928717 of the chimeric genome length
Sub genome represents 11.8935071283 of the chimeric genome length
$ ali2fasta.pl chimeric.ali
$ mv chimeric.fasta chimeric-bact.fasta
```

The chimeric genome `chimeric-bact.fasta` had 88.1% of Firmicutes CDS and 11.89% of (Gamma)Proteobacteria CDS, equally partitioned between redundant and non-redundant contaminations.

### 2.5 Chimeric eukaryotic genome creation

In order to demonstrate inter-domain contamination detection, we also created a eukaryotic genome including bacterial sequences. To this end, an uncontaminated eukaryotic genome was selected based on the analyses of [40], *Ustilago maydis* (`GCF_000328475.2`) (https://www.ncbi.nlm.nih.gov/assembly/GCF\_000328475.2/), a fungus belonging to Basidiomycota. This genome was then concatenated to the chimeric bacterial genome described above.

```
$ wget https://ftp.ncbi.nlm.nih.gov/genomes/all/GCF/000/328/475/GCF_000328475.2_Umaydis521_2.0/GCF_000328475.2_Umaydis521_2.0_genomic.fna.gz
$ gunzip GCF_000328475.2_Umaydis521_2.0_genomic.fna.gz
$ cat chimeric.fasta GCF_000328475.2_Umaydis521_2.0_genomic.fna > chimeric-euk-bact.fasta
```

Overall, this chimeric eukaryotic genome is composed of sequences belonging to Opisthokonta (80.1%), Firmicutes (16.1%) and (Gamma)Proteobacteria (3.8%).

## 3. Benchmarking

The five genomes (two reference bacteria, one chimeric bacterium, one reference eukaryote and one chimera between the eukaryote and the two bacteria) were analyzed with the six programs. Running times are reported for 20 CPU cores (AMD EPYC 7742 at 2.3 GHz), except for `Kraken2`, which was run on older hardware (Intel Xeon E5-2640 v4 at 2.4 GHz) due to taxonomic issues (see below).

### 3.1 Gene marker approaches

#### 3.1.1 CheckM [48]

```
# Command log for CheckM v1.1.3
$ mkdir chimeric
$ mv *.fasta chimeric/
$ checkm lineage_wf -t 20 -x fasta chimeric runa > checkm.result
```

`CheckM` took 9 minutes and 40 seconds.

```
# Results of CheckM
----------------------------------------------------------------------------------------------------------------------------------------------------------------------------------------------------
Bin Id                                            Marker lineage            # genomes   # markers   # marker sets    0     1     2    3    4    5+
Completeness   Contamination   Strain heterogeneity
----------------------------------------------------------------------------------------------------------------------------------------------------------------------------------------------------
Gammaproteobacteria_GCF_000007145.1     c__Gammaproteobacteria (UID4202)    67         481           276           1    480    0    0    0    0                 99.64            0.00               0.00
Firmicutes_GCF_000003645.1                 g__Bacillus (UID902)             44         1171          324           7    1163   1    0    0    0                 99.07            0.02               0.00
chimeric_bacterial                         g__Bacillus (UID902)             44         1171          324          104   1047   19   1    0    0                 91.79            1.65               0.00
Eukaryote_GCF_000328475.2                  k__Bacteria (UID203)            5449        104            58           38    34    20   12   0    0                 48.96           16.85               0.00
chimeric_eukaryotic                        k__Bacteria (UID203)            5449        101            56           2     12    36   33   13   5                 98.05           122.41              0.00
----------------------------------------------------------------------------------------------------------------------------------------------------------------------------------------------------
```

The output shows that no redundant contamination are found by `CheckM` in the two reference bacterial genomes. `CheckM` finds 1.65% of redundant contamination in the chimeric bacterial genome. This low level of contamination is a surprising result because the redundancy of the chimera is based on single-copy core genes, which have a higher chance of being picked-up by `CheckM`. `CheckM` finds 16.85% of redundant contamination in the reference eukaryotic genome, while labelling it as a bacterium. `CheckM` finds 122.41% of redundant contamination in the chimeric eukaryotic genome (including bacteria), while labelling it as a bacterium. The labelling of eukaryotes as bacteria is not surprising because `CheckM` was designed to work on bacteria only and can thus mistake mitochondria for bacteria, especially when (contaminating) Proteobacteria sequences are also present. Nevertheless, this demonstrates that a naive usage of `CheckM` on complex samples, composed of a mixture of eukaryotes and bacteria, can be difficult to interpret.

#### 3.1.2 EukCC [49]

```
# Command log for EukCC v2.1.0
$ wget http://ftp.ebi.ac.uk/pub/databases/metagenomics/eukcc/eukcc2_db_ver_1.1.tar.gz
$ tar -xzf eukcc2_db_ver_1.1.tar.gz
$ eukcc folder --out EUKCC --threads 20 chimeric/ --suffix .fasta --db eukcc2_db_ver_1.1
```

`EukCC` took 11 minutes and 12 seconds.

```
# Results of EukCC
bin                                             completeness    contamination
Gammaproteobacteria_GCF_000007145.1             0.0             0.0
Eukaryote_GCF_000328475.2                       100.0            0.0
chimeric_eukaryotic                             100.0           14.8
```

`EukCC` logically does not work on the two Firmicutes genomes (including the chimeric one) since it was designed for eukaryotes. In contrast, the (Gamma)Proteobacteria does pass in `EukCC`, which might be due to the (Alpha)Proteobacterial origin of mitochondria. However, both completeness and contamination values are null (0%), which indicates that this genome is not suitable for `EukCC`. The reference eukaryotic genome is not contaminated and complete, as expected. Regarding the chimeric eukaryotic genome, it shows a contamination level of 14.8%. This is higher than expected considering `EukCC` should only detect eukaryotic contaminants, whereas we introduced bacterial sequences.

#### 3.1.3 Forty-Two [36,42]

```
# Command log for 42 v0.213470

# setup
# download NCBI Taxonomy (also useful for Physeter below)
$ setup-taxdir.pl --taxdir=taxdump
# download ribosomal protein databases (in FASTA format)
$ git clone https://bitbucket.org/phylogeno/42-ribo-msas
# build BLAST databases for BRH reference organisms
$ cd 42-ribo-msas/ref_orgs/life/
$ for REFORG in *.fasta; do makeblastdb -in $REFORG \
    -dbtype prot -out `basename $REFORG .fasta` -parse_seqids; done
$ cd ../../../
# build BLAST databases for genomes to analyse
$ cd genomes/
$ for GENOME in *.fasta; do makeblastdb -in $GENOME \
    -dbtype nucl -out `basename $GENOME .fasta` -parse_seqids; done
$ cat > mapper-genomes.idm 
# Gammaproteobacteria_GCF_000007145.1 GCF_000007145.1_ASM714v1_genomic
# Firmicutes_GCF_000003645.1  GCF_000003645.1_ASM364v1_genomic
# chimeric_bacterial  chimeric-bact
# Eukaryote_GCF_000328475.2   GCF_000328475.2_Umaydis521_2.0_genomic
# chimeric_eukaryotic chimeric-bact-euk
# ^D
    
# build 42 YAML configuration file (using newer 'life' dataset)
$ yaml-generator-42.pl --run_mode=metagenomic \
    --out_suffix=-42 --queries 42-ribo-msas/queries/queries-life-fast.idl \
    --evalue=1e-3 --homologues_seg=yes --max_target_seqs=50 --templates_seg=no \
    --bank_dir genomes --bank_suffix=.nsq --bank_mapper genomes/mapper-genomes.idm --code=1 \
    --ref_brh=on --ref_bank_dir 42-ribo-msas/ref_orgs/life --ref_bank_suffix=.psq \
    --ref_bank_mapper 42-ribo-msas/ref_orgs/life/mapper-life-fast.idm \
    --ref_org_mul=0.33 --ref_score_mul=0.99 \
    --trim_homologues=on --trim_max_shift=5000 --trim_extra_margin=15 \
    --merge_orthologues=off --aligner_mode=off \
    --tax_reports=on --tax_dir taxdump \
    --tax_min_score=80 --tax_score_mul=0.95 --tax_min_ident=0 --tax_min_len=0 --tax_max_hits=10 \
    --tol_check=off
# run 42
$ forty-two.pl --config=config-42.yaml --outdir=reports \
    --verbosity=1 --threads=20 42-ribo-msas/MSAs/life/*.ali
# analyse 42 results
$ debrief-42.pl --indir=reports/ --in=-42 --taxdir=taxdump/ \
    --seq_labeling=42-ribo-msas/labelers/seq-labels.idl \
    --contam_labeling=42-ribo-msas/labelers/contam-labels.idl
```

`Forty-Two` took 5 mins and 1 sec.

```
# Results of 42
$ cut -f1-5,7-11 per-genome-42.stats | column -t
bank                                    tested_genes  added_ali  clean_ali  contam_ali  added_seq  clean_seq  contam_seq  unclass_contam_seq  unknown_seq
Gammaproteobacteria_GCF_000007145.1     98            52         52         0           52         52         0           0                   0
Firmicutes_GCF_000003645.1              98            47         47         0           47         47         0           0                   0
chimeric_bacterial                      98            48         42         40          82         42         40          0                   0
Eukaryote_GCF_000328475.2               98            58         47         6           67         51         6           0                   10
chimeric_eukaryotic                     98            84         47         48          148        51         87          0                   10

$ tail -n+2 chimeric_eukaryotic-42.tsv | sort -rnk1
51  SELF
40  cellular organisms; Bacteria; Terrabacteria group; Firmicutes; Bacilli; Bacillales; Bacillaceae; Bacillus; Bacillus cereus group
34  cellular organisms; Bacteria; Proteobacteria; Gammaproteobacteria; Xanthomonadales; Xanthomonadaceae; Xanthomonas
10  unknown
4   cellular organisms; Bacteria; Proteobacteria; Gammaproteobacteria; Xanthomonadales; Xanthomonadaceae; Xanthomonas; Xanthomonas campestris
2   cellular organisms; Bacteria; Proteobacteria
1   cellular organisms; Bacteria; Terrabacteria group; Firmicutes; Clostridia; Eubacteriales; Peptostreptococcaceae; Filifactor; Filifactor alocis; Filifactor alocis ATCC 35896
1   cellular organisms; Bacteria; Terrabacteria group; Firmicutes; Bacilli; Bacillales; Bacillaceae; Bacillus
1   cellular organisms; Bacteria; Proteobacteria; Gammaproteobacteria; Xanthomonadales; Xanthomonadaceae; Xanthomonas; Xanthomonas campestris; Xanthomonas campestris pv. campestris
1   cellular organisms; Bacteria; Proteobacteria; Gammaproteobacteria; Xanthomonadales; Xanthomonadaceae
1   cellular organisms; Bacteria; Proteobacteria; Alphaproteobacteria; Rickettsiales; Rickettsiaceae; Rickettsieae; Rickettsia
1   cellular organisms; Bacteria; Proteobacteria; Alphaproteobacteria; Rhodospirillales
1   cellular organisms; Bacteria; Proteobacteria; Alphaproteobacteria
0   unclassified
```

On the bacterial genomes, whether reference or chimeric, the inter-domain ‘life’ dataset composed of 98 ribosomal proteins performs exactly as the smaller prokaryotic (RiboDB) dataset composed of 90 proteins (data not shown). Both reference genomes are clean (0 `contam_ali` and 0 `contam_seq`). They are also rather complete, with 52 and 47 recovered single-copy ribosomal proteins (`added_ali`/`added_seq`) in the (Gamma)Proteobacteria and the Firmicutes, respectively. `Forty-Two` finds a lot of contaminants in the chimeric bacterial genome (40 `contam_ali`/`contam_seq`). This might be explained by an enrichment in ribosomal proteins among the single-copy core genes used to simulate the contamination. As the inter-domain dataset is still new, its performance is lower on the eukaryotic genomes in comparison to the well-tested eukaryotic dataset composed of 78 ribosomal proteins [40]. Indeed, some genuine eukaryotic ribosomal proteins are missed due to less sensitive BRH tests (47 vs 73 `clean_ali`, data not shown) whereas 6 “bacterial” homologues (`contam_ali`/`contam_seq`) are detected (and 10 remain tagged as `unknown_seq`); these mostly correspond to mitochondrial ribosomal proteins. Nevertheless, `Forty-Two` clearly reports that the chimeric eukaryotic genome is highly contaminated (48 `contam_ali` and 87 `contam_seq`). Moreover, the taxonomic analysis of the recovered ribosomal proteins globally matches the expectations, with `SELF` being *Ustilago*, and the other lineages corresponding to (Gamma)Proteobacteria (*Xanthomonas*) and Firmicutes (*Bacillus*), which are indeed the two bacterial genomes introduced in the fungal genome. Mitochondrial proteins appear clearly as (Alpha)Proteobacteria (and probably also as `unknown` entries).

### 3.2 Genome-wide approaches

#### 3.2.1 BLAST alignment

##### 3.2.1.1 GUNC [1]

```
# Command log for GUNC v1.0.5
$ gunc download_db guncDB
$ gunc run --db guncDB/gunc_db_progenomes2.1.dmnd \
    --input_fasta chimeric/GCF_000007145.1_ASM714v1_genomic.fasta --threads 20 \
    --out_dir GUNC/GCF_000007145.1
$ gunc run --db guncDB/gunc_db_progenomes2.1.dmnd \
    --input_fasta chimeric/GCF_000003645.1_ASM364v1_genomic.fasta --threads 20 \
    --out_dir GUNC/GCF_000003645.1
$ gunc run --db guncDB/gunc_db_progenomes2.1.dmnd \
    --input_fasta chimeric/chimeric-bact.fasta --threads 20 \
    --out_dir GUNC/chimeric-bact
$ gunc run --db guncDB/gunc_db_progenomes2.1.dmnd \
    --input_fasta chimeric/GCF_000328475.2_Umaydis521_2.0_genomic.fasta --threads 20 \
    --out_dir GUNC/GCF_000328475.2
$ gunc run --db guncDB/gunc_db_progenomes2.1.dmnd \
    --input_fasta chimeric/chimeric-bact-euk.fasta --threads 20 \
    --out_dir GUNC/chimeric-bact-euk
```

GUNC took 35 min and 10 seconds, requiring a large amount of time for the eukaryotic genomes but less than 3 minutes per bacterial genome.

```
# Results of GUNC
genome                                  n_genes_called  n_genes_mapped  n_contigs   taxonomic_level  proportion_genes_retained_in_major_clades       genes_retained_index    clade_separation_score  contamination_portion   n_effective_surplus_clades      mean_hit_identity       reference_representation_score  pass.GUNC
Gammaproteobacteria_GCF_000007145.1     4245            4198            1           kingdom 1.0      0.99    
0                       0.0                     0.0                     0.98                            0.97                    True
Firmicutes_GCF_000003645.1              5412            5307            1           kingdom 1.0      0.98    
0.0                      0.0                    0.0                     0.93                            0.91                    True
chimeric_bacterial                      5376            5293            5291        phylum  1.0      0.98     
1.0                      0.1                    0.23                    0.94                            0.92                    False
Eukaryote_GCF_000328475.2               11033           3117            25          kingdom 1.0      0.28    
0.0                     0.12                    0.27                    0.38                            0.11                    True
chimeric_eukaryotic                     16409           8411            5317        kingdom 1.0      0.51    
0.0                     0.04                    0.09                    0.73                            0.38                    True
```

GUNC detects no contamination in the two reference bacterial genomes, the contaminations scores being all at 0 while the `reference_representation_score` is high, indicating that both genomes map confidently to the database. The proportion of genes used is thus high, above 98%. The scores for the chimeric bacterial genome indicate that it maps correctly to the GUNC database with a `reference_representation_score` of 0.92 and 98% of genes used. The `contamination_portion` is 10%, which perfectly matches our simulations (11.89%). The reference eukaryotic genome shows few genes used, with only 28%, and its representation in the database is low too, 11%. This indicates that GUNC is not suitable for the evaluation of this genome, which was expected as GUNC was designed for bacteria. The delineation is more ambiguous for the eukaryotic genome contaminated by bacteria, since 51% of the genes are used with a `reference_representation_score` of 0.38. As above, this shows that a naive usage of such a detection program can be a source of errors.

##### 3.2.1.2 Physeter [3]

`Physeter` runs on BLAST reports. Here we used `DIAMOND blastx` [28] to generate them.  
The database used in this note is the inter-domain database constructed to work on complex samples [3]: see https://doi.org/10.3389/fmicb.2021.755101.

```
# Command log for Physeter v0.213470 (diamond v2.0.4.142)
$ mkdir temp

# Gammaproteobacteria_GCF_000007145.1
# mv GCF_000007145.1_ASM714v1_genomic.fna GCF_000007145.1.fasta
$ diamond blastx -d life-tqmd-of73.dmnd -q GCF_000007145.1.fasta \
    -o GCF_000007145.1.blastx -t temp -k 50 -e 1e-10 -f tab -p 20
$ physeter.pl GCF_000007145.1.blastx --fasta-dir=./ --outfile=contam_GCF_000007145.1.report \
    --taxdir=taxdump-20211206/ --taxon-list=contam-labels.idl 

# Firmicutes_GCF_000003645.1
# cp GCF_000003645.1_ASM364v1_genomic.fasta GCF_000003645.1.fasta
$ diamond blastx -d life-tqmd-of73.dmnd -q GCF_000003645.1.fasta \
    -o GCF_000003645.1.blastx  -t temp -k 50 -e 1e-10 -f tab -p 20
$ physeter.pl GCF_000003645.1.blastx --fasta-dir=./ --outfile=contam_GCF_000003645.1.report \
    --taxdir=taxdump-20211206/ --taxon-list=contam-labels.idl

# chimeric_bacterial 
$ diamond blastx -d life-tqmd-of73.dmnd -q chimeric-bact.fasta \
    -o chimeric-bact.blastx -t temp -k 50 -e 1e-10 -f tab -p 20
$ physeter.pl chimeric-bact.blastx --fasta-dir=./ --outfile=contam_chim.report \
    --taxdir=taxdump-20211206/ --taxon-list=contam-labels.idl \
    --auto-detect --tax-min-hits=1 --tax-max-hits=50 --kfold=life-tqmd-of73.gca

# Eukaryote_GCF_000328475.2
# mv GCF_000328475.2_Umaydis521_2.0_genomic.fasta GCF_000328475.2.fasta
$ diamond blastx -d life-tqmd-of73.dmnd -q GCF_000328475.2.fasta \
    -o GCF_000328475.2.blastx -t temp -k 50 -e 1e-10 -f tab -p 20
$ physeter.pl GCF_000328475.2.blastx --fasta-dir=./ --outfile=contam_GCF_000328475.2.report \
    --taxdir=taxdump-20211206/ --taxon-list=contam-labels.idl \
    --exp-tax=Opisthokonta 

# chimeric_eukaryotic
$ diamond blastx -d life-tqmd-of73.dmnd -q chimeric-bact-euk.fasta \
    -o chimeric-bact-euk.blastx -t temp -k 50 -e 1e-10 -f tab -p 20
$ physeter.pl chimeric-bact-euk.blastx --fasta-dir=./ --outfile=contam_chimeukal-lca.report \
    --taxdir=taxdump-20211206/ --taxon-list=contam-labels.idl \
    --auto-detect --tax-min-hits=1 --tax-max-hits=50
$ physeter.pl chim-bact-euk-split.blastx --fasta-dir=./ --outfile=contam_chimeukal-lca.report \
    --taxdir=taxdump-20211206/ --taxon-list=contam-labels.idl \
    --auto-detect --tax-min-hits=1 --tax-max-hits=50 --kfold=life-tqmd-of73.gca
```

Altogether, `DIAMOND blastx` and `Physeter` took 1 hour, 22 minutes and 1 second.

```
# Results of Physeter
# Gammaproteobacteria_GCF_000007145.1 with default options
Gammaproteobacteria_GCF_000007145.1 Proteobacteria  100.00  0.00    0.00    0.00            1.00

# Firmicutes_GCF_000003645.1 with default options
Firmicutes_GCF_000003645.1           Firmicutes      100.00  0.00    0.00    0.00            1.00

# chimeric bacterial, kfold and auto-detection
chimeric_bacterial   Firmicutes      21.79   25.77   11.77   40.67   Proteobacteria=9.88,Terrabacteria group=4.16,Euryarchaeota=2.92,Opisthokonta=1.89,Synergistetes=1.67,Thermotogae=1.02,TACK group=0.83,PVC group=0.52,Spirochaetes=0.50,Viridiplantae=0.50,FCB group=0.31,Stramenopiles=0.31,Amoebozoa=0.26,Alveolata=0.22,Aquificae=0.22,Cryptophyceae=0.17,Haptista=0.11,Rhodophyta=0.11,Rhizaria=0.07,Glaucocystophyceae=0.06,Euglenozoa=0.04 2.90
chimeric-bacterial   Firmicutes      21.94   25.27   12.21   40.58   Proteobacteria=9.10,Terrabacteria group=3.66,Euryarchaeota=2.89,Opisthokonta=2.02,Synergistetes=1.59,Thermotogae=1.05,TACK group=0.93,Viridiplantae=0.65,PVC group=0.50,Spirochaetes=0.48,Thermodesulfobacteria=0.44,Amoebozoa=0.35,FCB group=0.35,Stramenopiles=0.31,Alveolata=0.24,Aquificae=0.19,Cryptophyceae=0.19,Haptista=0.11,Rhodophyta=0.11,Rhizaria=0.06,Glaucocystophyceae=0.04,Euglenozoa=0.02      2.89
chimeric_bacterial   Firmicutes      21.52   25.98   11.86   40.64   Proteobacteria=9.88,Terrabacteria group=3.53,Euryarchaeota=3.26,Opisthokonta=1.98,Synergistetes=1.67,Thermotogae=0.98,TACK group=0.91,Viridiplantae=0.52,PVC group=0.48,Thermodesulfobacteria=0.46,Spirochaetes=0.44,Amoebozoa=0.35,FCB group=0.35,Stramenopiles=0.31,Alveolata=0.20,Cryptophyceae=0.19,Aquificae=0.17,Haptista=0.11,Rhodophyta=0.07,Rhizaria=0.06,Glaucocystophyceae=0.04,Euglenozoa=0.02      2.84
chimeric_bacterial   Firmicutes      22.85   24.83   11.71   40.61   Proteobacteria=9.94,Terrabacteria group=3.96,Euryarchaeota=3.05,Synergistetes=1.65,TACK group=1.02,Thermotogae=0.96,Opisthokonta=0.59,Viridiplantae=0.57,Spirochaetes=0.48,Amoebozoa=0.41,PVC group=0.41,Thermodesulfobacteria=0.41,FCB group=0.30,Stramenopiles=0.30,Aquificae=0.19,Cryptophyceae=0.17,Haptista=0.13,Alveolata=0.09,Glaucocystophyceae=0.07,Rhodophyta=0.07,Rhizaria=0.06,Euglenozoa=0.02      2.98
chimeric_bacterial   Firmicutes      22.55   25.07   11.82   40.56   Proteobacteria=9.99,Terrabacteria group=3.39,Euryarchaeota=2.79,Opisthokonta=1.81,Synergistetes=1.61,Thermotogae=1.00,TACK group=0.81,Viridiplantae=0.52,PVC group=0.46,Spirochaetes=0.46,Thermodesulfobacteria=0.41,FCB group=0.33,Amoebozoa=0.31,Alveolata=0.24,Stramenopiles=0.24,Aquificae=0.17,Cryptophyceae=0.15,Haptista=0.11,Rhodophyta=0.11,Rhizaria=0.09,Glaucocystophyceae=0.04,Euglenozoa=0.02      2.94
chimeric_bacterial   Firmicutes      22.33   25.42   11.95   40.30   Proteobacteria=9.90,Terrabacteria group=3.68,Euryarchaeota=2.81,Opisthokonta=1.96,Synergistetes=1.61,Thermotogae=0.94,TACK group=0.81,Viridiplantae=0.56,PVC group=0.48,Spirochaetes=0.44,Thermodesulfobacteria=0.41,FCB group=0.37,Stramenopiles=0.35,Amoebozoa=0.31,Alveolata=0.20,Aquificae=0.17,Cryptophyceae=0.17,Haptista=0.11,Rhodophyta=0.06,Euglenozoa=0.04,Glaucocystophyceae=0.04    2.92
chimeric_bacterial   Firmicutes      22.33   25.37   11.54   40.76   Proteobacteria=9.75,Terrabacteria group=4.26,Euryarchaeota=3.02,Opisthokonta=1.89,Synergistetes=1.05,TACK group=0.83,Thermotogae=0.83,Viridiplantae=0.61,PVC group=0.52,Thermodesulfobacteria=0.48,FCB group=0.43,Spirochaetes=0.39,Stramenopiles=0.33,Amoebozoa=0.28,Alveolata=0.22,Cryptophyceae=0.17,Haptista=0.11,Rhodophyta=0.11,Rhizaria=0.06,Glaucocystophyceae=0.04     2.97
chimeric_bacterial   Firmicutes      22.70   25.05   11.88   40.37   Proteobacteria=9.42,Terrabacteria group=3.70,Euryarchaeota=2.96,Opisthokonta=1.94,Synergistetes=1.74,TACK group=0.85,Thermotogae=0.81,Viridiplantae=0.54,PVC group=0.48,Thermodesulfobacteria=0.43,Amoebozoa=0.39,FCB group=0.39,Stramenopiles=0.33,Alveolata=0.22,Aquificae=0.17,Cryptophyceae=0.17,Spirochaetes=0.17,Haptista=0.11,Rhodophyta=0.11,Rhizaria=0.06,Euglenozoa=0.04,Glaucocystophyceae=0.04      2.87
chimeric_bacterial   Firmicutes      21.83   25.53   12.04   40.60   Proteobacteria=9.25,Terrabacteria group=3.81,Euryarchaeota=3.20,Opisthokonta=2.04,Synergistetes=1.61,Thermotogae=0.98,TACK group=0.81,Viridiplantae=0.56,PVC group=0.52,Spirochaetes=0.46,Thermodesulfobacteria=0.44,FCB group=0.41,Stramenopiles=0.30,Alveolata=0.26,Cryptophyceae=0.22,Amoebozoa=0.19,Aquificae=0.19,Haptista=0.11,Rhodophyta=0.09,Rhizaria=0.06,Euglenozoa=0.04      2.88
chimeric_bacterial   Firmicutes      22.37   25.20   11.91   40.52   Proteobacteria=9.45,Terrabacteria group=3.98,Euryarchaeota=3.20,Opisthokonta=2.00,Synergistetes=1.68,TACK group=0.93,Thermotogae=0.63,Spirochaetes=0.48,FCB group=0.46,Thermodesulfobacteria=0.46,Stramenopiles=0.37,Viridiplantae=0.37,Amoebozoa=0.31,Alveolata=0.24,Aquificae=0.22,PVC group=0.13,Rhodophyta=0.13,Rhizaria=0.06,Glaucocystophyceae=0.04,Cryptophyceae=0.02,Euglenozoa=0.02,Haptista=0.02      2.92

# Eukaryote_GCF_000328475.2 with Opisthokonta as an expected taxon
Eukaryote_GCF_000328475.2 Opisthokonta              100.00  0.00    0.00    0.00            1.00

# chimeric eukaryotic genome, auto-detection, min 2 hits
chimeric_eukaryotic       Firmicutes      22.09   26.09   11.67   40.15   Proteobacteria=9.78,Terrabacteria group=3.68,Euryarchaeota=3.22,Opisthokonta=2.37,Synergistetes=1.64,Thermotogae=0.94,TACK group=0.79,Viridiplantae=0.63,Thermodesulfobacteria=0.50,PVC group=0.48,Spirochaetes=0.39,FCB group=0.35,Stramenopiles=0.33,Amoebozoa=0.31,Alveolata=0.22,Cryptophyceae=0.17,Haptista=0.11,Rhodophyta=0.09,Glaucocystophyceae=0.04,Rhizaria=0.04,Euglenozoa=0.02     2.87

# chimeric eukaryotic genome, after splitting, kfold and auto-detection
chimeric_eukaryotic     Opisthokonta    56.63   2.94    0.84    39.59   Firmicutes=1.42,Proteobacteria=0.62,Euryarchaeota=0.20,Actinobacteria=0.13,Synergistetes=0.10,Terrabacteria group=0.10,Thermotogae=0.06,TACK group=0.06,Viridiplantae=0.03,PVC group=0.03,Spirochaetes=0.03,Thermodesulfobacteria=0.03,Amoebozoa=0.02,Bacteroidetes=0.02,Stramenopiles=0.02,Alveolata=0.02,Aquificae=0.01,Rhodophyta=0.01,Haptista=0.01,Rhizaria=0.00,Cryptophyceae=0.00,Euglenozoa=0.00,Glaucocystophyceae=0.00        1.16
chimeric_eukaryotic     Opisthokonta    56.62   2.94    0.85    39.59   Firmicutes=1.41,Proteobacteria=0.60,Euryarchaeota=0.20,Actinobacteria=0.12,Terrabacteria group=0.12,Synergistetes=0.10,Thermotogae=0.06,TACK group=0.06,Viridiplantae=0.04,PVC group=0.03,Spirochaetes=0.03,Thermodesulfobacteria=0.03,Bacteroidetes=0.03,Stramenopiles=0.02,Amoebozoa=0.02,Alveolata=0.02,Cryptophyceae=0.01,Aquificae=0.01,Rhodophyta=0.01,Haptista=0.01,Euglenozoa=0.00,Glaucocystophyceae=0.00,Rhizaria=0.00        1.16
chimeric_eukaryotic     Opisthokonta    56.54   2.99    0.86    39.61   Firmicutes=1.43,Proteobacteria=0.63,Euryarchaeota=0.20,Actinobacteria=0.14,Terrabacteria group=0.13,Synergistetes=0.09,Thermotogae=0.07,TACK group=0.06,Viridiplantae=0.04,PVC group=0.03,Spirochaetes=0.03,Thermodesulfobacteria=0.03,Bacteroidetes=0.02,Stramenopiles=0.02,Amoebozoa=0.02,Alveolata=0.01,Cryptophyceae=0.01,Aquificae=0.01,Haptista=0.01,Rhodophyta=0.01,Euglenozoa=0.00,Glaucocystophyceae=0.00,Rhizaria=0.00        1.16
chimeric_eukaryotic     Opisthokonta    56.62   2.99    0.79    39.60   Firmicutes=1.46,Proteobacteria=0.64,Euryarchaeota=0.18,Actinobacteria=0.14,Terrabacteria group=0.14,Synergistetes=0.09,TACK group=0.06,Thermotogae=0.05,Viridiplantae=0.04,PVC group=0.03,Bacteroidetes=0.03,Amoebozoa=0.02,Stramenopiles=0.02,Aquificae=0.02,Alveolata=0.01,Cryptophyceae=0.01,Haptista=0.01,Spirochaetes=0.01,Euglenozoa=0.01,Rhodophyta=0.01,Glaucocystophyceae=0.00,Rhizaria=0.00   1.16
chimeric_eukaryotic     Opisthokonta    56.63   2.92    0.86    39.59   Firmicutes=1.45,Proteobacteria=0.56,Euryarchaeota=0.20,Actinobacteria=0.12,Terrabacteria group=0.11,Synergistetes=0.11,Thermotogae=0.06,TACK group=0.05,PVC group=0.03,Spirochaetes=0.03,Thermodesulfobacteria=0.03,Amoebozoa=0.02,Bacteroidetes=0.02,Viridiplantae=0.02,Stramenopiles=0.02,Alveolata=0.02,Cryptophyceae=0.01,Aquificae=0.01,Haptista=0.01,Rhodophyta=0.00,Rhizaria=0.00,Euglenozoa=0.00,Glaucocystophyceae=0.00        1.16
chimeric_eukaryotic     Opisthokonta    56.62   2.96    0.82    39.60   Firmicutes=1.45,Proteobacteria=0.64,Euryarchaeota=0.18,Actinobacteria=0.12,Terrabacteria group=0.12,Synergistetes=0.10,TACK group=0.06,Thermotogae=0.06,Viridiplantae=0.04,PVC group=0.03,Spirochaetes=0.03,Thermodesulfobacteria=0.03,Stramenopiles=0.02,Bacteroidetes=0.02,Amoebozoa=0.02,Alveolata=0.02,Cryptophyceae=0.01,Aquificae=0.01,Haptista=0.01,Rhodophyta=0.01,Rhizaria=0.00,Euglenozoa=0.00,Glaucocystophyceae=0.00        1.16
chimeric_eukaryotic     Opisthokonta    56.63   2.94    0.82    39.61   Firmicutes=1.44,Proteobacteria=0.62,Euryarchaeota=0.16,Actinobacteria=0.14,Terrabacteria group=0.13,Synergistetes=0.11,TACK group=0.06,Thermotogae=0.04,Viridiplantae=0.04,Spirochaetes=0.03,Thermodesulfobacteria=0.03,Stramenopiles=0.03,Bacteroidetes=0.02,PVC group=0.02,Amoebozoa=0.02,Alveolata=0.02,Aquificae=0.01,Cryptophyceae=0.01,Rhodophyta=0.01,Euglenozoa=0.00,Rhizaria=0.00,Glaucocystophyceae=0.00,Haptista=0.00        1.17
chimeric_eukaryotic     Opisthokonta    56.63   2.89    0.86    39.62   Firmicutes=1.35,Proteobacteria=0.64,Euryarchaeota=0.21,Actinobacteria=0.13,Terrabacteria group=0.12,Synergistetes=0.10,Thermotogae=0.07,TACK group=0.06,Spirochaetes=0.03,Viridiplantae=0.03,Thermodesulfobacteria=0.03,Bacteroidetes=0.03,Stramenopiles=0.02,Alveolata=0.02,Amoebozoa=0.01,Aquificae=0.01,Cryptophyceae=0.01,PVC group=0.01,Haptista=0.01,Rhodophyta=0.01,Glaucocystophyceae=0.00,Euglenozoa=0.00,Rhizaria=0.00        1.15
chimeric_eukaryotic     Opisthokonta    9.95    3.18    2.10    84.77   Firmicutes=1.45,Proteobacteria=0.63,Euryarchaeota=0.19,Actinobacteria=0.13,Terrabacteria group=0.12,Synergistetes=0.11,Stramenopiles=0.07,Thermotogae=0.07,Viridiplantae=0.06,Amoebozoa=0.06,TACK group=0.05,PVC group=0.04,Spirochaetes=0.03,Thermodesulfobacteria=0.03,Rhodophyta=0.03,Rhizaria=0.02,Haptista=0.02,Alveolata=0.02,Bacteroidetes=0.02,Cryptophyceae=0.01,Euglenozoa=0.01,Glaucocystophyceae=0.01       2.62
chimeric_eukaryotic     Opisthokonta    56.63   2.97    0.82    39.58   Firmicutes=1.45,Proteobacteria=0.64,Euryarchaeota=0.21,Actinobacteria=0.11,Synergistetes=0.10,Terrabacteria group=0.10,Thermotogae=0.06,TACK group=0.05,Viridiplantae=0.03,PVC group=0.03,Thermodesulfobacteria=0.03,Bacteroidetes=0.02,Spirochaetes=0.02,Amoebozoa=0.02,Stramenopiles=0.02,Alveolata=0.02,Cryptophyceae=0.01,Aquificae=0.01,Haptista=0.01,Rhodophyta=0.01,Rhizaria=0.00,Euglenozoa=0.00
```

`Physeter` identifies no contamination in the three reference genomes in auto-detection mode, as expected since it can work in an inter-domain setting. On the chimeric bacterial genome, `Physeter` correctly identifies the main organism (as Firmicutes) and Proteobacteria as the main contaminant, with (as expected) nearly 10%. However `Physeter` overestimates the contamination level by detecting other contaminants that are supposedly not present in the genome. When the bacterial and eukaryotic genomes are concatenated, `Physeter` detects the Firmicutes as the main organism. This is a logical result since we used CDS as sequences for bacteria, which represent thousands of individual sequences compared to the 37 scaffolds of the genuine eukaryotic genome. Indeed `Physeter` reports its estimates in terms of sequence numbers (whether CDS, genome contigs or scaffolds, reads etc). When the eukaryotic genome is split into pseudo-reads, as recommended in Cornet et al. (2018) [22] and Lupo et al. (2021) [3], `Physeter` correctly identifies the eukaryotic genome as the main organism.

#### 3.2.2 Long k-mer matching

##### 3.2.2.1 Kraken2 [55]

At the time of writing, we were not able to install the `Kraken2` database, probably because of a recent modification in the architecture of the NCBI Taxonomy. Since `Kraken` (and `Kraken2`) have been maintained over the last ten years, we have no doubt that the current issue will be addressed by the authors. Meanwhile, we used the `nt` database installed (a couple of weeks earlier) on our older computing cluster. Therefore, the running time reported here should be interpreted as an upper bound only.

```
# Command log for Kraken2 v2.0.8-beta
$ kraken2 --use-names --db /media/vol1/databases/kraken2-nt/ \
     GCF_000007145.1_ASM714v1_genomic.fasta --threads 40 \
     --report GCF_000007145.1_ASM714v1_genomic.report > GCF_000007145.1_ASM714v1_genomic.kraken
$ kraken2 --use-names --db /media/vol1/databases/kraken2-nt/ \
     GCF_000003645.1_ASM364v1_genomic.fasta --threads 40 \
     --report GCF_000003645.1_ASM364v1_genomic.report > GCF_000003645.1_ASM364v1_genomic.kraken
$ kraken2 --use-names --db /media/vol1/databases/kraken2-nt/ \
    chimeric-bact.fasta --threads 40 \
    --report chimeric-bact.report > chimeric-bact.kraken
$ kraken2 --use-names --db /media/vol1/databases/kraken2-nt/ \
    GCF_000328475.2_Umaydis521_2.0_genomic.fasta --threads 40 \
    --report GCF_000328475.2_Umaydis521_2.0_genomic.report > GCF_000328475.2_Umaydis521_2.0_genomic.kraken
$ kraken2 --use-names --db /media/vol1/databases/kraken2-nt/ \
    GCF_000328475.2-split.fasta --threads 40 \
    --report GCF_000328475.2-split.report > GCF_000328475.2-split.kraken
$ kraken2 --use-names --db /media/vol1/databases/kraken2-nt/ \
    chimeric-bact-euk-split.fasta --threads 40 \
    --report chimeric-bact-euk-split.report > chimeric-bact-euk-split.kraken
```

`Kraken2` on the `nt` database took 5 hours, 51 minutes and 12 seconds.

```
# Gammaproteobacteria_GCF_000007145.1
100.00  1       0       R       1       root
100.00  1       0       R1      131567    cellular organisms
100.00  1       0       D       2           Bacteria
100.00  1       0       P       1224          Proteobacteria
100.00  1       0       C       1236            Gammaproteobacteria
100.00  1       0       O       135614            Xanthomonadales
100.00  1       0       F       32033               Xanthomonadaceae
100.00  1       0       G       338                   Xanthomonas
100.00  1       0       S       339                     Xanthomonas campestris
100.00  1       0       S1      340                       Xanthomonas campestris pv. campestris
100.00  1       1       S2      190485                      Xanthomonas campestris pv. campestris str. ATCC 33913

# Firmicutes_GCF_000003645.1
100.00  1       0       R       1       root
100.00  1       0       R1      131567    cellular organisms
100.00  1       0       D       2           Bacteria
100.00  1       0       D1      1783272       Terrabacteria group
100.00  1       0       P       1239            Firmicutes
100.00  1       0       C       91061             Bacilli
100.00  1       0       O       1385                Bacillales
100.00  1       0       F       186817                Bacillaceae
100.00  1       0       G       1386                    Bacillus
100.00  1       0       G1      86661                     Bacillus cereus group
100.00  1       1       S       2026186                     Bacillus paranthracis

# chimeric_bacterial
0.20  11      11      U       0       unclassified
99.80  5394    26      R       1       root
99.30  5367    6       R1      131567    cellular organisms
99.04  5353    7       D       2           Bacteria
88.86  4803    2       D1      1783272       Terrabacteria group
88.83  4801    0       P       1239            Firmicutes
88.81  4800    0       C       91061             Bacilli
88.81  4800    0       O       1385                Bacillales
88.68  4793    437     F       186817                Bacillaceae
80.52  4352    364     G       1386                    Bacillus
73.45  3970    1576    G1      86661                     Bacillus cereus group
21.44  1159    1159    S       2026186                     Bacillus paranthracis
11.40  616     505     S       1396                        Bacillus cereus
0.78  42      42      S1      361100                        Bacillus cereus Q1
0.43  23      23      S1      405535                        Bacillus cereus AH820
0.43  23      0       S1      1179100                       Bacillus cereus biovar anthracis
0.43  23      23      S2      637380                          Bacillus cereus biovar anthracis str. CI 
...
10.05  543     0       P       1224          Proteobacteria
10.05  543     0       C       1236            Gammaproteobacteria
9.95  538     0       O       135614            Xanthomonadales
9.95  538     0       F       32033               Xanthomonadaceae
9.95  538     5       G       338                   Xanthomonas
9.86  533     230     S       339                     Xanthomonas campestris
5.57  301     280     S1      340                       Xanthomonas campestris pv. campestris
0.31  17      17      S2      190485                      Xanthomonas campestris pv. campestris str. ATCC 33913
0.04  2       2       S2      1358004                     Xanthomonas campestris pv. campestris str. CN03
0.02  1       1       S2      1281283                     Xanthomonas campestris pv. campestris str. CN15
0.02  1       1       S2      1358018                     Xanthomonas campestris pv. campestris str. CN13
0.04  2       0       S1      359385                    Xanthomonas campestris pv. raphani
0.04  2       2       S2      990315                      Xanthomonas campestris pv. raphani 756C
0.07  4       0       O       91347             Enterobacterales
0.06  3       0       F       543                 Enterobacteriaceae
0.06  3       0       F1      2890311               Klebsiella/Raoultella group
0.06  3       0       G       570                     Klebsiella
0.04  2       2       S       2058152                   Klebsiella grimontii
0.02  1       1       S       548                       Klebsiella aerogenes
...

# Eukaryote_GCF_000328475.2
100.00  27      0       R       1       root
100.00  27      0       R1      131567    cellular organisms
100.00  27      0       D       2759        Eukaryota
100.00  27      0       D1      33154         Opisthokonta
100.00  27      0       K       4751            Fungi
100.00  27      0       K1      451864            Dikarya
100.00  27      0       P       5204                Basidiomycota
100.00  27      0       P1      452284                Ustilaginomycotina
100.00  27      0       C       5257                    Ustilaginomycetes
100.00  27      0       O       5267                      Ustilaginales
100.00  27      0       F       5268                        Ustilaginaceae
100.00  27      0       G       5269                          Ustilago
100.00  27      0       S       5270                            Ustilago maydis
100.00  27      27      S1      237631                            Ustilago maydis 521

# Eukaryote_GCF_000328475.2, with split in pseudo-reads
24.92  19597   19597   U       0       unclassified
75.08  59045   18      R       1       root
75.02  59001   260     R1      131567    cellular organisms
74.21  58363   121     D       2759        Eukaryota
73.78  58021   135     D1      33154         Opisthokonta
71.19  55988   2       K       4751            Fungi
71.18  55981   19      K1      451864            Dikarya
70.98  55821   5       P       5204                Basidiomycota
70.96  55802   5       P1      452284                Ustilaginomycotina
70.94  55791   0       C       5257                    Ustilaginomycetes
70.94  55791   0       O       5267                      Ustilaginales
70.94  55791   109     F       5268                        Ustilaginaceae
70.42  55376   3       G       5269                          Ustilago
70.35  55328   3044    S       5270                            Ustilago maydis
66.48  52280   52280   S1      237631                            Ustilago maydis 521
0.01  4       4       S1      559306                            Ustilago maydis FB1
0.05  43      43      S       307758                          Ustilago bromivora
0.00  1       1       S       120017                          Ustilago hordei
0.00  1       1       S       185366                          Ustilago esculenta
0.22  170     21      G       63265                         Sporisorium
0.09  70      44      S       72558                           Sporisorium reilianum
0.02  15      15      S1      999809                            Sporisorium reilianum SRZ2
0.01  11      11      S1      72559                             Sporisorium reilianum f. sp. reilianum
0.09  69      69      S       49012                           Sporisorium scitamineum
0.01  10      10      S       280036                          Sporisorium graminicola
...

# chimeric_eukaryotic
23.33  19608   19608   U       0       unclassified
76.67  64439   44      R       1       root
76.59  64368   266     R1      131567    cellular organisms
69.45  58371   121     D       2759        Eukaryota
69.04  58028   135     D1      33154         Opisthokonta
66.62  55988   2       K       4751            Fungi
66.61  55981   19      K1      451864            Dikarya
66.42  55821   5       P       5204                Basidiomycota
66.39  55802   5       P1      452284                Ustilaginomycotina
66.38  55791   0       C       5257                    Ustilaginomycetes
66.38  55791   0       O       5267                      Ustilaginales
66.38  55791   109     F       5268                        Ustilaginaceae
65.89  55376   3       G       5269                          Ustilago
65.83  55328   3044    S       5270                            Ustilago maydis
62.20  52280   52280   S1      237631                            Ustilago maydis 521
0.00  4       4       S1      559306                            Ustilago maydis FB1
0.05  43      43      S       307758                          Ustilago bromivora
0.00  1       1       S       120017                          Ustilago hordei
0.00  1       1       S       185366                          Ustilago esculenta
0.20  170     21      G       63265                         Sporisorium
0.08  70      44      S       72558                           Sporisorium reilianum
0.02  15      15      S1      999809                            Sporisorium reilianum SRZ2
66.42  55821   5       P       5204                Basidiomycota
66.39  55802   5       P1      452284                Ustilaginomycotina
66.38  55791   0       C       5257                    Ustilaginomycetes
66.38  55791   0       O       5267                      Ustilaginales
66.38  55791   109     F       5268                        Ustilaginaceae
65.89  55376   3       G       5269                          Ustilago
65.83  55328   3044    S       5270                            Ustilago maydis
62.20  52280   52280   S1      237631                            Ustilago maydis 521
 0.00  4       4       S1      559306                            Ustilago maydis FB1
...
6.81  5724    25      D       2           Bacteria
5.85  4917    3       D1      1783272       Terrabacteria group
5.77  4852    0       P       1239            Firmicutes
5.75  4834    0       C       91061             Bacilli
5.73  4819    0       O       1385                Bacillales
5.71  4799    437     F       186817                Bacillaceae
5.18  4354    364     G       1386                    Bacillus
4.72  3970    1576    G1      86661                     Bacillus cereus group
1.38  1159    1159    S       2026186                     Bacillus paranthracis
0.73  616     505     S       1396                        Bacillus cereus
0.05  42      42      S1      361100                        Bacillus cereus Q1
0.03  23      23      S1      405535                        Bacillus cereus AH820
0.03  23      0       S1      1179100                       Bacillus cereus biovar anthracis
0.03  23      23      S2      637380                          Bacillus cereus biovar anthracis str. CI
...
0.86  727     8       P       1224          Proteobacteria
0.75  634     3       C       1236            Gammaproteobacteria
0.66  557     0       O       135614            Xanthomonadales
0.66  555     1       F       32033               Xanthomonadaceae
0.65  543     7       G       338                   Xanthomonas
0.63  533     230     S       339                     Xanthomonas campestris
0.36  301     280     S1      340                       Xanthomonas campestris pv. campestris
0.02  17      17      S2      190485                      Xanthomonas campestris pv. campestris str. ATCC 33913
...
```

`Kraken2` finds no contamination in the reference genomes. As for `Physeter`, the results are computed based on the number of sequences. When the eukaryotic genome is split into pseudo-reads, the number of unclassified sequences increases and the kmer affiliation change. `Kraken2` correctly identifies the main organism in the chimeric bacterial genome, with 88% of Firmicutes and 10% of Proteobacteria, as expected from the simulated data. `Kraken2` works also correctly in our inter-domain setting, even if the proportions of each organism are not well estimated (69.5% of Eukaryota vs 80.1% in the simulation, 6.1% of Firmicutes vs 16.1% in the simulation, 0.86% of Proteobacteria vs 3.8% in the simulation).

(END)
